# Supplementary material for: PROP1 triggers epithelial-mesenchymal transition-like process in pituitary stem cells
Source: eLife. 2016 Jun 28;5:e14470. doi: 10.7554/eLife.14470 (PMC4940164; doi:10.7554/eLife.14470)
Supplement: Supplementary file 1. — (A) Primary antibodies used in this study. (B) Secondary Antibodies in this study. (C) Primers for RT-PCR used to confirm ChIP-Seq peaks. DOI: http://dx.doi.org/10.7554/eLife.14470.018 [file elife-14470-supp1.docx]

**SI EXPERIMENTAL PROCEDURES**

**Supplementary file 1A: Primary antibodies used in this study:**

| **Name** | **Species** | **dilution** | **Source** |
| --- | --- | --- | --- |
| SOX2 | Goat | 1:100 | Neuromics, GT15098 |
| PROP1 | guinea pig | 1:200 | Gift from Aimee Ryan (McGill University, Montréal) |
| POU1F1 | Rabbit | 1:100 | Gift from S. Rhodes (Indiana University) |
| CYCLIN E | Rabbit | 1:200 | Santa Cruz, sc481 |
| CYCLIND1 | Mouse monoclonal | 1:200 | Santa Cruz, sc8396 |
| CYCLIND2 | Rabbit | 1:200 | Santa Cruz, sc593 |
| p27^kip1^ | Mouse monoclonal | 1:100 | Millipore, NA35 |
| p57^kip2^ | Mouse monoclonal | 1:100 | Thermo Fisher, MS897B1 |
| CDH1 or  E-CADHERIN | Mouse monoclonal | 1:100 | BD Biosciences, 610181 |
| CDH2 or N-CADHERIN | Rabbit | 1:100 | Abcam, ab12221 |
| GFRa2 | Rabbit | 1:70 | Abcam, ab8027 |

**Supplementary file 1B: Secondary Antibodies used at 1:100 dilution:**

Anti-rabbit biotinylated, donkey, Jackson Immunoresearch, 711066152

Anti-goat biotinylated, donkey, Life Technologies, D20698

Anti-mouse biotinylated, sheep, Jackson Immunoresearch, 515-065-003

Anti-guinea pig biotinylated, donkey, Jackson Immunoresearch, 706065148

**Supplementary file 1C: Primers for RT-PCR used to confirm ChIP-Seq peaks:**

| **Gene (Fw, forward; Rv reverse)** | **DNA sequence (5’ to 3’)** |
| --- | --- |
| Pou1f1 (peak *) Fw | TTGAATGTTCAGGCTCTCATT |
| Pou1f1 (peak *) Rv | TTAGCTGCTATCTCGAACTTGA |
| Pou1f1 (peak **) Fw | ACACTGCTGTGCCTGAGTTA |
| Pou1f1 (peak **) Rv | ACACTGCTGTGCCTGAGTTA |
| Gli2 Fw | CATCACCACCACACACACTT |
| Gli2 Rv | TGTGGTGTGGCATAAATACG |
| Notch2 Fw | GATAGGGAAGTAGAGTGCCATC |
| Notch2 Rv | CTATTAATGCCAGCTTTGTGAG |
| Zeb2 Fw | GTCAGAACGCAACCTCACTT |
| Zeb2 Rv | CCTGGCAGCCTTACCTTTAT |
| Cldn23 Fw | TGCTGCCACCTACAGACTAA |
| Cldn23 Rv | CTTTGGTTCATCTGCAACAG |
